# Hypertension and dyslipidemia are risk factors for herpes zoster in patients with rheumatoid arthritis: a retrospective analysis using a medical information database

**DOI:** 10.1007/s00296-021-04889-1

**Published:** 2021-06-06

**Authors:** Keiko Tanaka, Eizen Kimura, Kensuke Oryoji, Shin-ichi Mizuki, Tomoko Kobayashi, Atsushi Nishikawa, Eiko Yoshinaga, Yoshihiro Miyake

**Affiliations:** 1 Department of Epidemiology and Preventive Medicine, Ehime University Graduate School of Medicine, Shitsukawa, Toon, Ehime 791-0295 Japan; 2 Research Promotion Unit, Translation Research Center, Ehime University Hospital, Ehime, Japan; 3 Center for Data Science, Ehime University, Ehime, Japan; 4 National Institute of Public Health, Saitama, Japan; 5 Department of Medical Informatics, Ehime University Graduate School of Medicine, Ehime, Japan; 6 Center for Rheumatic Diseases, Matsuyama Red Cross Hospital, Matsuyama, Ehime Japan; 7 Medicines Development Unit Japan and Medical Affairs, Eli Lilly Japan K.K, Hyogo, Japan

**Keywords:** Arthritis, Rheumatoid, Dyslipidemias, Herpes zoster, Hypertension, Incidence

## Abstract

This study used data from a large-scale multicenter medical information database in Japan to estimate the incidence rate of herpes zoster (HZ) and to examine the relationship between hypertension, dyslipidemia, and diabetes mellitus (DM), and the risk of HZ among patients with rheumatoid arthritis (RA). The research dataset consisted of 221,196 records of potential target patients with RA extracted between April 1, 2008 and August 31, 2017 from the Medical Data Vision database. To assess the association between hypertension, dyslipidemia, and DM and the risk of HZ, a case–control study was set up. Records of 101,498 study subjects met the inclusion criteria. During the observation period, 2566 patients developed HZ and the overall incidence rate was 5.2 (95% confidence interval: 5.0–5.4 per 1000 patient-years). Hypertension, dyslipidemia, and DM were significantly associated with an increased risk of HZ after adjustment for sex, age, hospital size, and use of anti-rheumatic drugs. When mutual adjustment was made for hypertension, dyslipidemia, and DM, the positive associations between hypertension and dyslipidemia and the risk of HZ remained significant; however, the positive association with DM completely disappeared. RA patients with hypertension or dyslipidemia may be at higher risk of HZ.

**Supplementary Information:**

The online version contains supplementary material available at 10.1007/s00296-021-04889-1.

## Introduction

Rheumatoid arthritis (RA) is one of the most prevalent chronic inflammatory diseases and an autoimmune disease that affects the synovial joints [1]. In Japan, the incidence rate was 0.08 per 1000 person-years between 1985 and 1996 [2]. The prevalence apparently declined between 1965 and 1996 (2.4 to 1.7 per 1000 persons) [2]. Although the etiology of RA is still unclear, it is accepted as a multifactorial disease. Genetic and environmental factors, and their interactions, might contribute to the development and expression of the disease [3].

Herpes zoster (HZ) is caused by the reactivation of the latent varicella-zoster virus dormant in the sensory ganglia [4]. HZ is known to be more frequent in patients with conditions that depress cell-mediated immunity such as malignancy, immunosuppressive disorders, and treatment with immunosuppressants [5]. In a previous study using a large US database and UK database, the risk of HZ was increased twofold in patients with RA compared with patients without RA [6]. A US population-based cohort study also showed that the cumulative incidence of HZ was higher in the RA patients than the non-RA subjects between 1980 and 2008: the cumulative incidence rates of HZ for RA patients and non-RA subjects were 12.1 and 5.4 per 1000 person-years, respectively [7]. In Japan, a cohort study of RA patients reported that the incidence rate of HZ was 6.66 per 1000 person-years during 5-year follow-up until 2013 [8]. According to a recent systematic review in 2019 in patients with autoimmune inflammatory rheumatic diseases, the pooled incidence rate of HZ among RA patients was 11.6 per 1000 person-years [9]. The medications used to treat RA that modulate the immune system might affect the development of HZ in patients with RA. In studies concerning the risk factors for HZ among patients with RA, RA medications such as biological disease-modifying anti-rheumatic drugs (bDMARDs), corticosteroids, or methotrexate (MTX) have been compared for their effect on the development of HZ [7, 10, 11]. Recently, RA management has dramatically changed [12, 13]. Such alteration of treatment strategies might affect clinical circumstances in RA patients, including the risk of developing HZ. Newer information is needed in regard to this issue.

Besides immunosuppressive conditions, advanced age, female sex, and white skin are considered as potential risk factors for HZ; however, there is still a lack of information regarding other risk factors for HZ [14]. Some previous studies indicated that chronic medical conditions contribute to the development of HZ [15–17]. Because chronic medical conditions such as hypertension, dyslipidemia, and diabetes mellitus (DM) are widely prevalent in RA patients [18, 19], it is possible that those chronic medical conditions might also be risk factors for HZ in patients with RA. To develop prevention strategies for HZ in the future in subjects with suppressed immunity, it is meaningful to identify conditions that might affect the development of HZ. Currently, other than RA-related factors such as disease activity or medications, only advanced age is identified as a risk factor for HZ among RA patients [7–10, 20, 21].

The purpose of this study was to estimate the incidence rate of HZ in patients with RA using a large-scale multicenter medical information database in Japan. In addition, we aimed to examine the associations between three medical conditions (hypertension, dyslipidemia, and DM) and the risk of HZ in patients with RA.

## Methods

### Data source

The present study was carried out using the medical database managed by Medical Data Vision (MDV) Co., Ltd., in Tokyo, Japan. The commercial health database included records of 19 million patients as of August 2017. The data from participating healthcare institutions were anonymized before transfer to MDV. The database is composed of integrated medical claims, pharmacy claims, and Diagnosis Procedure Combination (DPC) data [22], and includes the data of both inpatients and outpatients from about 18% of all acute medical care hospitals in Japan.

For the present study, the research dataset (*n* = 221,196) consisted of the records (including a disease history sheet and a clinical action sheet) of potential target patients (patients with the Tenth Revision of the International Statistical Classification of Diseases and Related Health Problems [ICD-10] codes related to RA: M05 and M06) extracted between April 1, 2008 and August 31, 2017 from the MDV database. The study protocol was approved by the ethics committee of Ehime University Graduate School of Medicine (No. 1709001). According to the Ethical Guideline for Epidemiological Research issued by the Japanese Ministry of Health, Labor and Welfare, written informed consent does not apply to anonymized data. Therefore, the requirement for written informed consent was waived for this analysis.

### Study population

In the present study, when patients in our research dataset had the first RA diagnosis on or after April 1, 2008, the earlier of the two dates (A) and (B) was defined as the index date: (A) the date of the first RA diagnosis and (B) the date of the first prescription of anti-rheumatic drugs (except NSAIDs, anti-rheumatic medicine are listed in Supplementary file, Table S1). Our research dataset also included patients who had the first RA diagnosis before April 1, 2008. In such patients, the index day was set at April 1, 2008 (C). However, because the date of birth was not recorded in the disease history sheet, age on the index day was unknown regardless of the index date (A), (B), or (C). To identify the patient’s age on the index day, the year and month of birth of the patients was estimated using the prescription history in clinical action sheets collected between April 1, 2008 and August 31, 2017. Since a clinical action sheet included the age at the beginning of the month at the time of issuance of the medical fee claim, the month of birth could be identified as the month in which the age on the clinical action sheet increased by 1 year. Nevertheless, 833 patients whose age could not be estimated were excluded from the present study.

Patients were included if they met all the following criteria: (1) having an estimated age at index date of ≥ 18 years; (2) having a follow-up period of at least 180 days after the index date prior to the last date of data availability; (3) having at least two outpatient diagnoses of RA (ICD-10 codes: M05 or M06) during the follow-up period.

### Study variables

HZ occurrence was defined as positive if patients were prescribed an anti-viral drug (anti-viral drugs are listed in Supplementary file, Table S2) within 2 weeks before or after a diagnosis or suspicion of HZ (ICD-10 code B02).

In the present study, as possible risk factors of HZ among patients with RA, we included three medical conditions of interest: hypertension, dyslipidemia, and DM. Hypertension was defined as present if patients had a prescription for drugs with indication for hypertension (hypertension drugs are listed in Supplementary file, Table S3). Dyslipidemia or DM was also defined as positive if patients had a prescription for drugs with indication for dyslipidemia or DM, respectively (dyslipidemia and DM drugs are listed in Supplementary file, Tables S4 and S5, respectively). Use of corticosteroid was based on records of prescription medications, which are listed in Supplementary file (Table S6). Daily doses of prednisolone (or equivalent dose) were defined as the maximum daily dose prescribed during the follow-up period. Dose of steroids was categorized into < 5, and ≥ 5 mg/day.

### Statistical analysis

The incidence rate of HZ was calculated as the number of events per 1000 patient-years. Logistic regression models were used to assess the association between medical conditions and the risk of HZ. In the multivariate analysis, the odds ratios (ORs) were adjusted for age at the index date, sex, hospital size (< 200, 200–499, and ≥ 500 beds), and use of conventional synthetic DMARDs (csDMARDs), bDMARDs, targeted synthetic DMARDs (tsDMARDs), MTX, and steroids (< 5 and ≥ 5 mg/day). All statistical analyses were performed using the SAS software package version 9.4 (SAS Institute, Inc., Cary, NC, USA). Two-sided *p* values < 0.05 were considered statistically significant.

## Results

Of the 221,196 records of patients identified in the MDV database, the records of 101,498 patients were included in the study after applying the above-mentioned inclusion criteria. Table 1 shows the characteristics of the study population. The mean age was 61.9 years (standard deviation: 14.0 years). The proportion of women was 71.5%. In the study population, 34,186 (33.7%), 19,593 (19.3%), and 10,642 (10.5%) patients had hypertension, dyslipidemia, and DM, respectively. The proportion of patients who were prescribed csDMARDs, MTX, and ≥ 5 mg/day corticosteroids was 46.6%, 50.0%, and 47.3%, respectively. Compared with patients without HZ (controls), patients with HZ (cases) were more likely to be older, to have hypertension, dyslipidemia, and DM, and to be using tsDMARDs and steroids. The proportion of patients by hospital size was similar in the two groups.

**Table 1:** Characteristics of study population

	*n* (%) or mean ± SD			Crude OR (95% CI)
	Overall (*n* = 101,498)	Herpes zoster		
		Cases (*n* = 2566)	Controls (*n* = 98,932)	
*Subjects*				
Age, years	61.9 ± 14.0	62.7 ± 13.0	61.8 ± 14.0	1.01 (1.00–1.01)
Sex				
Men	28,952 (28.5)	656 (25.6)	28,296 (28.6)	1.00
Women	72,546 (71.5)	1910 (74.4)	70,636 (71.4)	0.17 (1.07–1.28)
Hospital size, beds				
< 200	9530 (9.4)	236 (9.2)	9294 (9.4)	1.00
200–499	43,212 (42.6)	1118 (43.6)	42,094 (42.6)	1.05 (0.91–1.21)
≥ 500	48,756 (48.0)	1212 (47.2)	47,544 (48.1)	1.00 (0.87–1.16)
Medical condition				
Hypertension	34,186 (33.7)	1101 (42.9)	33,085 (33.4)	1.50 (1.38–1.62)
Dyslipidemia	19,593 (19.3)	621 (24.2)	18,972 (19.2)	1.35 (1.23–1.47)
Diabetes mellitus	10,642 (10.5)	334 (13.0)	10,308 (10.4)	1.35 (1.23–1.47)
Anti-rheumatic drug used				
csDMARDs	47,334 (46.6)	995 (38.8)	46,339 (46.8)	0.72 (0.66–0.78)
bDMARDs	18,949 (18.7)	478 (18.6)	18,471 (18.7)	1.00 (0.90–1.10)
tsDMARDs	429 (0.42)	19 (0.74)	410 (0.41)	1.79 (1.09–2.76)
MTX	50,734 (50.0)	983 (38.3)	49,751 (50.3)	0.61 (0.57–0.67)
Steroids (mg/day)				
< 5	53,491 (52.7)	1066 (41.5)	52,425 (53.0)	1.00
≥ 5	48,007 (47.3)	1500 (58.5)	46,507 (47.0)	1.59 (1.47–1.72)

*bDMARDs* biological disease-modifying anti-rheumatic drugs; *CI* confidence interval; *csDMARDs* conventional synthetic disease-modifying anti-rheumatic drugs; *MTX* methotrexate; *OR* odds ratio; *SD* standard deviation; *tsDMARDs* targeted synthetic disease-modifying anti-rheumatic drugs

In the overall population of 101,498 patients, 2,566 patients developed HZ during their observation period: incidence rate was 5.2 (95% confidence interval (CI): 5.0–5.4 per 1000 patient-years) (Table 2). Incidence rates of HZ for men and women were 5.2 (95% CI: 4.9–5.7 per 1000 patient-years) and 5.1 (95% CI: 4.9–5.4 per 1000 patient-years), respectively. The incidence rates by age group indicated the lowest in the 30–39-year age group (3.8 per 1000 patient-years), a stepwise increase to the highest in the 70–79-year age group (6.1 per 1000 patient-years), and then a decrease in the 80 years and older age group (5.9 per 1000 patient-years).

**Table 2:** Incidence rates of HZ by age and sex among RA patients, Japan

Age group	No. of events	Patients-years	Incidence rate/1000 patient-years (95% CI)
Overall (*n* = 101,498)			
< 30	54	11,459	4.7 (3.6–6.2)
30–39	111	29,329	3.8 (3.1–4.6)
40–49	218	52,639	4.1 (3.6–4.7)
50–59	485	110,932	4.4 (4.0–4.8)
60–69	846	151,638	5.6 (5.2–6.0)
70–79	693	114,387	6.1 (5.6–6.5)
≥ 80	159	26,991	5.9 (5.0–6.9)
Total	2566	497,375	5.2 (5.0–5.4)
Male (*n* = 28,952)			
< 30	10	2440	4.1 (2.2–7.6)
30–39	20	5832	3.4 (2.2–5.3)
40–49	49	10,753	4.6 (3.4–6.0)
50–59	113	25,447	4.4 (3.7–5.3)
60–69	230	41,214	5.6 (4.9–6.4)
70–79	194	32,083	6.0 (5.3–7.0)
≥ 80	40	7357	5.4 (4.0–7.4)
Total	656	125,125	5.2 (4.9–5.7)
Female (*n* = 72,546)			
< 30	44	9020	4.9 (3.6–6.6)
30–39	91	23,497	3.9 (3.2–4.8)
40–49	169	41,886	4.0 (3.5–4.7)
50–59	372	85,485	4.4 (3.9–4.8)
60–69	616	110,424	5.6 (5.2–6.0)
70–79	499	82,304	6.1 (5.6–6.6)
≥ 80	119	19,634	6.1 (5.1–7.3)
Total	1910	372,250	5.1 (4.9–5.4)

*CI* confidence interval; *HZ* herpes zoster; *RA* rheumatoid arthritis

Table 3 shows crude and adjusted ORs and 95% CIs for the risk of HZ in relation to hypertension, dyslipidemia, and DM. In the crude analysis, hypertension, dyslipidemia, and DM were significantly associated with an increased risk of HZ. After adjustment for sex, age, hospital size, and use of csDMARDs, bDMARDs, tsDMARDs, MTX, and steroids, a positive association between those medical conditions and risk of HZ was attenuated, but remained significant: the adjusted ORs (95% CIs) for hypertension, dyslipidemia, and DM were 1.33 (1.22–1.44), 1.22 (1.11–1.33), and 1.16 (1.03–1.30), respectively.

**Table 3:** Odds ratios and 95% confidence intervals of HZ among RA patients, Japan

	*n* (%)		Adjusted OR (95% CI)^*^	Mutual adjusted OR (95% CI)^†^
	Cases (*N* = 2,566)	Controls (*N* = 98,932)		
Hypertension				
No	1465 (57.1)	65,847 (66.6)	1.00	1.00
Yes	1101 (42.9)	33,085 (33.4)	1.33 (1.22–1.44)	1.28 (1.18–1.40)
Dyslipidemia				
No	1945 (75.8)	79,960 (80.8)	1.00	1.00
Yes	621 (24.2)	18,972 (19.2)	1.22 (1.11–1.33)	1.11 (1.004–1.22)
Diabetes mellitus				
No	2232 (89.8)	88,624 (89.6)	1.00	1.00
Yes	334 (13.0)	10,308 (10.4)	1.16 (1.03–1.30)	1.04 (0.92–1.18)

*CI* confidence interval; *HZ* herpes zoster; *OR* odds ratio; *RA* rheumatoid arthritis

^*^Adjusted for sex, age, hospital size, and use of csDMARDs, bDMARDs, tsDMARDs, MTX, and steroids

^†^Adjusted for sex, age, hospital size, use of csDMARDs, bDMARDs, tsDMARDs, MTX, and steroids, hypertension, dyslipidemia, and diabetes mellitus

When mutual adjustment was made for hypertension, dyslipidemia, and DM, the positive associations between hypertension and dyslipidemia and the risk of HZ remained significant; however, the significant association with DM completely disappeared: the mutually adjusted ORs (95% CIs) for hypertension, dyslipidemia, and DM were 1.28 (1.18–1.40), 1.11 (1.004–1.22), and 1.04 (0.92–1.18), respectively.

## Discussion

In this study using a large-scale multicenter medical information database in Japan, the incidence rate of HZ in patients with RA was 5.2 per 1000 person-years, which was lower than those reported in previous studies in RA patients [6, 21, 23]. A large observational cohort of Japanese RA patients reported that the incidence of HZ was 12.1 per 1000 person-years between 2005 and 2010 [21]. A 5-year prospective cohort study of Japanese RA patients reported an incidence rate of 6.66 per 1000 person-years [8]. In studies from Western countries using the data of the Veterans Affairs healthcare system [23] and the US PharMetrics claims database [6], the reported incidence rates of HZ in patients with RA were 9.99 and 9.83 per 1000 person-years, respectively. The relatively lower incidences of HZ in the present study are likely to be mainly ascribable to the strict definition of HZ. In the present study, we defined patients as HZ positive when they were prescribed an anti-viral drug within 2 weeks before or after a diagnosis or suspicion of HZ (ICD-10 code B02). Among other previous studies on incidence rate, there were various definitions of HZ, such as confirmation by medical chart review or audit following patient-reporting [21], clinical diagnosis by investigators [8], and ICD-9-CM code [15] or OXMIS and Read codes [6]. Therefore, it is not possible to compare the results among the above-mentioned studies directly. When HZ cases in the present study were defined as a diagnosis or suspicion of HZ using ICD-10 code B02 only, without data on anti-viral drugs, the incidence rate was 10.1 (95% CI: 9.8–10.4 per 1000 person-years), which appears to be similar to those reported in previous studies.

The present study examined three chronic conditions as possible risk factors for HZ among RA patients and revealed that hypertension and dyslipidemia, but not DM, were independently associated with the development of HZ. To date, no study examined the influence of chronic medical conditions on the risk of HZ in RA patients. Some case–control studies in RA patients showed that, compared with patients without HZ, patients with HZ more frequently had DM [6, 11], whereas other case–control studies showed that DM was slightly less frequent among patients with HZ [8, 24]. These cited studies, however, did not assess whether DM was related to the risk of HZ among RA patients. Regarding hypertension and dyslipidemia, even the difference in distributions in the RA patients with and without HZ has never been previously reported.

Using commercial claims and encounters data, Joesoef et al. [15] previously explored whether common chronic medical conditions affect the development of HZ in the general population, and showed that DM and hyperlipidemia, but not hypertension, were risk factors for HZ [15]. On the other hand, a Japanese retrospective hospital-based cohort study revealed that patients with DM or hypertension had a significantly higher risk of HZ than patients with other underlying diseases [25]. Other studies showed that patients with type II DM and statin users were also at higher risk of HZ [16, 17]. In a meta-analysis conducted in 2017 [26], DM and statin use are listed as risk factors for HZ, whereas hypertension is not mentioned. Results of these studies regarding the influence of DM on HZ are inconsistent with our finding of no association between DM and HZ.

An advantage of this study is the use of a large-size database. However, the present study also has some limitations. Data on rheumatoid factor and anticyclic citrullinated peptide antibody, which are used for RA diagnosis, were not available in the present study, preventing us from clarifying the accuracy of RA patient identification. However, our study patients were identified by their having had at least two outpatient diagnoses of RA and their taking of anti-rheumatic medicine. A US validation study of the ICD-10 codes for RA (M05 and M06) showed that they were likely to be reasonably useful in identifying RA patients even when laboratory data were not available [27]. Additionally, Kim et al. reported that by the use of a combination of at least two or more RA diagnosis codes and DMARD prescriptions, it is possible to accurately identify RA patients in a health care utilization database [28]. Thus, it is unlikely that patients without RA were included in the study, although no validation study of the MDV database methodology or search algorithms was conducted. For the MDV database, it is not possible to link data from different institutions. Therefore, if a patient had consultations in different hospitals, the present data would be incomplete. For example, if RA patients with HZ received HZ treatment at a medical institution other than the institution providing RA treatment, this would lead to an underestimation of the incidence of HZ. Although the MDV database included ICD-10 codes, the codes are recorded for the purpose of payment. Thus, it is possible that some misclassifications of diseases occurred. To increase the specificity of outcome classification, in the present study, we defined the development of HZ as prescription of an anti-viral drug within 2 weeks before or after a diagnosis or suspicion of HZ. However, outcome misclassification cannot be avoided, and such nondifferential outcome misclassification would have manifested as an underestimation of observed associations. In the present study, because the MDV database included laboratory data for only approximately 10% of patients, we were not able to use such data to define hypertension, dyslipidemia, and DM. The definitions of hypertension, dyslipidemia, and DM were based on only the prescriptions of drugs with indication for those diseases. Patients are likely to receive care for those diseases in different medical institutions, leading to an underestimation of exposures. Such nondifferential exposure misclassification would tend to reduce the observed association. Although age, sex, and hospital size were controlled in the multivariate model, potential important confounders such as smoking, alcohol intake, and physical activity, which might influence the association between hypertension, dyslipidemia, and DM and the risk of HZ, were not taken into consideration. Furthermore, a lack of information regarding the degree of disease activity, such as disease activity score, would have significant impact on the observed associations. It is possible that patients with higher disease activity might be more susceptible to HZ than those with lower disease activity. Although the MDV database includes hospital-based medical and pharmacy claims data from a large number of large hospitals throughout Japan, our results cannot be generalized to all patients with RA in Japan, because patients who were included in the MDV database might be different from patients who consulted in medical institutions other than large hospitals. Therefore, when interpreting our results, the differences between DPC hospitals and clinics need to be taken into consideration. In the present study, only 101,498 patients of the 221,196 identified in the MDV database were included in the analysis (45.9%). Compared with RA patients in another Japanese observational cohort study (Institute of Rheumatology, Rheumatoid Arthritis, IORRA) [11], our study patients were more likely to be older (61.9 vs 58.9 years), to be men (28.5% vs 16.0%), and to use steroids (66.6% vs 53.1%). Using a large-scale medical database in Japan, we were able to quantify the incidence rates of HZ according to age and gender in patients with RA.

Despite its many limitations, the present study suggests that hypertension and dyslipidemia may be associated with an increased risk of HZ in patients with RA. Additional studies are needed to confirm our findings, to clarify mechanisms underlying the observed associations, and to identify preventive methods for the HZ development among RA patients with certain chronic medical conditions.

## Supplementary Information

Below is the link to the electronic supplementary material.

Supplementary file1 (DOCX 26 KB)

## Data Availability

The data underlying this article cannot be shared without permission from the owner, which is Medical Data Vision (MDV) Co. Ltd.
