# Supplementary material for: Hypertension and dyslipidemia are risk factors for herpes zoster in patients with rheumatoid arthritis: a retrospective analysis using a medical information database
Source: Rheumatol Int. 2021 Jun 6;41(9):1633–9. doi: 10.1007/s00296-021-04889-1 (PMC8316192; doi:10.1007/s00296-021-04889-1)
Supplement: Supplementary file 1 — Supplementary file1 (DOCX 26 KB) [file 296_2021_4889_MOESM1_ESM.docx]

Supplementary table 1. List of anti-rheumatic drugs

| **No.** | **Generic name** | **Category** |
| --- | --- | --- |
| 1 | Tacrolimus Hydrate | csDMARD |
| 2 | Mizoribine | csDMARD |
| 3 | Iguratimod | csDMARD |
| 4 | Bucillamine | csDMARD |
| 5 | leflunomide | csDMARD |
| 6 | Azathioprine | csDMARD |
| 7 | Ciclosporine | csDMARD |
| 8 | Sodium aurothiomalate | csDMARD |
| 9 | Hydroxychloroquine | csDMARD |
| 10 | Salazosul fapyridine | csDMARD |
| 11 | Leflunomide | csDMARD |
| 12 | Mycophenolate mofetil, mycophenolic acid | csDMARD |
| 13 | Cyclophosphamide | csDMARD |
| 14 | D-penicillamine | csDMARD |
| 15 | Auranofin | csDMARD |
| 16 | Tofacitinib citrate | tsDMARD |
| 17 | Tocilizumab | bDMARD |
| 18 | Etanercept | bDMARD |
| 19 | Adalimumab | bDMARD |
| 20 | Abatacept | bDMARD |
| 21 | Infliximab | bDMARD |
| 22 | Certolizumab | bDMARD |
| 23 | Golimumab | bDMARD |
| 24 | Methotrexate | MTX |

Supplementary table 2. List of targeted anti-viral drugs

| **No.** | **Generic name** |
| --- | --- |
| 1 | ‎Aciclovir |
| 2 | Valaciclovir hydrochloride |

Supplementary table 3. List of dyslipidemia drugs

| **No.** | **Generic name** |
| --- | --- |
| 1 | Bezafibrate |
| 2 | Clofibrate |
| 3 | Clinofibrate |
| 4 | Fenofibrate |
| 5 | Gamma Oryzanol |
| 6 | Icosapent ethyl |
| 7 | Niceritrol |
| 8 | Nicomol |
| 9 | Polyenephosphatidyl choline |
| 10 | Pravastatin sodium |
| 11 | Probucol |
| 12 | Simvastatin |
| 13 | Tocopherol nicotinate |
| 14 | Lomitapide Mesilate |
| 15 | Ezetimibe |
| 16 | Pemafibrate |
| 17 | γ-Oryzanol |

Supplementary table 4. List of hypertension drug

| **No.1** | **Generic Name** |
| --- | --- |
| 1 | Acebutolol Hydrochloride |
| 2 | Alacepril |
| 3 | Aliskiren Fumarate |
| 4 | Amlodipine Besilate |
| 5 | Amosulalol Hydrochloride |
| 6 | Aranidipine |
| 7 | Arotinolol Hydrochloride |
| 8 | Atenolol |
| 9 | Azelnidipine |
| 10 | Azilsartan |
| 11 | Barnidipine Hydrochloride |
| 12 | Benzylhydrochlorothiazide |
| 13 | Betaxolol Hydrochloride |
| 14 | Bisoprolol |
| 15 | Bisoprolol Fumarate |
| 16 | Bunazosin Hydrochloride |
| 17 | Candesartan Cilexetil |
| 18 | Candesartan Cilexetil/Amlodipine Besilate Combined Drug |
| 19 | Captopril |
| 20 | Carteolol Hydrochloride |
| 21 | Carvedilol |
| 22 | Celiprolol Hydrochloride |
| 23 | Cilazapril Hydrate |
| 24 | Cilnidipine |
| 25 | Clonidine Hydrochloride |
| 26 | Delapril Hydrochloride |
| 27 | Diltiazem Hydrochloride |
| 28 | Doxazosin Mesilate |
| 29 | Efonidipine Hydrochloride Ethanolate |
| 30 | Enalapril Maleate |
| 31 | Eplerenone |
| 32 | Felodipine |
| 33 | Furosemide |
| 34 | Guanabenz Acetate |
| 35 | Hydralazine Hydrochloride |
| 36 | Hydrochlorothiazide |
| 37 | Imidapril Hydrochloride |
| 38 | Indapamide |
| 39 | Irbesartan |
| 40 | Irbesartan/Amlodipine Besilate Combined Drug |
| 41 | Irbesartan/Trichlormethiazide Combined Drug |
| 42 | Labetalol Hydrochloride |
| 43 | Lisinopril Hydrate |
| 44 | Losartan Potassium |
| 45 | Losartan Potassium/Hydrochlorothiazide Combined Drug |
| 46 | Manidipine Hydrochloride |
| 47 | Mefruside |
| 48 | Methyldopa Hydrate |
| 49 | Meticrane |
| 50 | Metoprolol Tartrate |
| 51 | Nadolol |
| 52 | Nicardipine Hydrochloride |
| 53 | Nifedipine |
| 54 | Nilvadipine |
| 55 | Nipradilol |
| 56 | Nisoldipine |
| 57 | Nitrendipine |
| 58 | Olmesartan Medoxomil |
| 59 | Olmesartan Medoxomil/Azelnidipine Combined Drug |
| 60 | Perindopril Erbumine |
| 61 | Pindolol |
| 62 | Prazosin Hydrochloride |
| 63 | Prazosin Hydrochloride |
| 64 | Propranolol Hydrochloride |
| 65 | Quinapril Hydrochloride |
| 66 | Reserpine |
| 67 | Sodium Nitroprusside Hydrate |
| 68 | Spironolactone |
| 69 | Telmisartan |
| 70 | Telmisartan/Amlodipine Besilate Combined Drug |
| 71 | Telmisartan/Amlodipine Besilate/Hydrochlorothiazide Combined Drug |
| 72 | Telmisartan/Hydrochlorothiazide Combined Drug |
| 73 | Temocapril Hydrochloride |
| 74 | Terazosin Hydrochloride Hydrate |
| 75 | Trandolapril |
| 76 | Triamterene |
| 77 | Trichlormethiazide |
| 78 | Tripamide |
| 79 | Urapidil |
| 80 | Valsartan |
| 81 | Valsartan/Amlodipine Besilate Combined Drug |
| 82 | Valsartan/Cilnidipine Combined Drug |
| 83 | Valsartan/Hydrochlorothiazide Combined Drug |

Supplementary table 5. List of diabetes medicine

| **No.** | **Generic name** |
| --- | --- |
| 1 | Voglibose |
| 2 | Acarbose |
| 3 | Pioglitazone Hydrochloride |
| 4 | Tofogliflozin Hydrate |
| 5 | Chlorpropamide |
| 6 | Glimepiride |
| 7 | Vildagliptin |
| 8 | Metformin Hydrochloride |
| 9 | Glibenclamide |
| 10 | Saxagliptin Hydrate |
| 11 | Canagliflozin Hydrate |
| 12 | Sitagliptin Phosphate Hydrate |
| 13 | Gliclazide |
| 14 | Metformin Hydrochloride |
| 15 | Mitiglinide Calcium Hydrate |
| 16 | Chlorpropamide |
| 17 | Trelagliptin Succinate |
| 18 | Buformin Hydrochloride |
| 19 | Acetohexamide |
| 20 | Empagliflozin |
| 21 | Repaglinide |
| 22 | Anagliptin |
| 23 | Ipragliflozin L-Proline |
| 24 | Nateglinide |
| 25 | Miglitol |
| 26 | Glyclopyramide |
| 27 | Linagliptin |
| 28 | Alogliptin benzoate |
| 29 | Omarigliptin |
| 30 | Dapagliflozin Propylene Glycolate Hydrate |
| 31 | Luseogliflozin Hydrate luseogliflozin |

Supplementary table 6. List of Steroids

| **No.** | **Generic name** |
| --- | --- |
| 1 | Betamethason |
| 2 | Prednisolone |
| 3 | Dexamethasone sodium |
